# 人尿液*N*-糖蛋白/*N*-糖肽规模化富集鉴定

**DOI:** 10.3724/SP.J.1123.2021.01039

**Published:** 2021-07-08

**Authors:** Shiting SHANG, Hangyan DONG, Yuanyuan LI, Wanjun ZHANG, Hang LI, Weijie QIN, Xiaohong QIAN

**Affiliations:** 军事科学院军事医学研究院生命组学研究所, 北京蛋白质组研究中心, 蛋白质组学国家重点实验室, 北京 102206; Institute of Lifeomics, Academy of Military Medical Sciences, Academy of Military Sciences, Beijing Proteome Research Center, State Key Laboratory of Proteomics, Beijing 102206, China; 军事科学院军事医学研究院生命组学研究所, 北京蛋白质组研究中心, 蛋白质组学国家重点实验室, 北京 102206; Institute of Lifeomics, Academy of Military Medical Sciences, Academy of Military Sciences, Beijing Proteome Research Center, State Key Laboratory of Proteomics, Beijing 102206, China; 军事科学院军事医学研究院生命组学研究所, 北京蛋白质组研究中心, 蛋白质组学国家重点实验室, 北京 102206; Institute of Lifeomics, Academy of Military Medical Sciences, Academy of Military Sciences, Beijing Proteome Research Center, State Key Laboratory of Proteomics, Beijing 102206, China; 军事科学院军事医学研究院生命组学研究所, 北京蛋白质组研究中心, 蛋白质组学国家重点实验室, 北京 102206; Institute of Lifeomics, Academy of Military Medical Sciences, Academy of Military Sciences, Beijing Proteome Research Center, State Key Laboratory of Proteomics, Beijing 102206, China; 军事科学院军事医学研究院生命组学研究所, 北京蛋白质组研究中心, 蛋白质组学国家重点实验室, 北京 102206; Institute of Lifeomics, Academy of Military Medical Sciences, Academy of Military Sciences, Beijing Proteome Research Center, State Key Laboratory of Proteomics, Beijing 102206, China; 军事科学院军事医学研究院生命组学研究所, 北京蛋白质组研究中心, 蛋白质组学国家重点实验室, 北京 102206; Institute of Lifeomics, Academy of Military Medical Sciences, Academy of Military Sciences, Beijing Proteome Research Center, State Key Laboratory of Proteomics, Beijing 102206, China; 军事科学院军事医学研究院生命组学研究所, 北京蛋白质组研究中心, 蛋白质组学国家重点实验室, 北京 102206; Institute of Lifeomics, Academy of Military Medical Sciences, Academy of Military Sciences, Beijing Proteome Research Center, State Key Laboratory of Proteomics, Beijing 102206, China

**Keywords:** 亲水相互作用色谱法, 生物质谱, 糖蛋白质组, *N*-糖肽, 富集, hydrophilic interaction chromatography (HILIC), biological mass spectrometry, glycoproteome, *N*-glycopeptide, enrichment

## Abstract

蛋白质的*N*-糖基化是真核细胞中一种重要的翻译后修饰,*N*-糖基化修饰在调控细胞黏附、迁移、信号转导及细胞凋亡等方面扮演着关键角色。蛋白质糖基化修饰的异常变化与多种重要疾病的发生相关。尿液具有蛋白质组复杂程度低和非入侵性等特点,适合大量及连续多时间点采样研究。但由于个体差异和生理条件的影响,尿蛋白丰度的生理波动较大。目前缺乏对健康人群尿液*N*-糖蛋白的个体差异和生理波动的专门性研究,以及生理丰度范围的构建,难以将个体差异、正常生理波动和疾病导致的变化进行有效区分,对疾病标志物研究提出很大挑战。本研究以亲水相互作用色谱法(HILIC)为基础,对该富集方法中活化、清洗与洗脱过程进行优化,其中主要对HILIC填料粒径和富集缓冲体系进行优化,并考察了不同实验条件下*N*-糖肽富集的鉴定数量、选择性与稳定性,发现当HILIC填料粒径为5 μm,在三氟乙酸富集体系下有更高的*N*-糖蛋白/*N*-糖肽鉴定水平。在此基础上,对20例健康男性志愿者和20例健康女性志愿者的尿液*N*-糖蛋白/*N*-糖肽进行了富集和定性、定量及功能分析。从40例尿液样本中共鉴定到1016个*N*-糖蛋白、2192条*N*-糖肽。采用非标定量策略对尿液*N*-糖肽的生理丰度波动范围进行了考察,尿液*N*-糖肽的丰度跨度约5个数量级。在此之后探索了健康人群尿蛋白*N*-糖基化水平的性别差异,筛选出性别相关的差异*N*-糖蛋白后进行了功能分析。统计学分析显示在尿液样本中性别可能是产生个体差异的重要因素。该工作为基于尿液糖蛋白质组学的功能与机制研究和临床生物标志物筛选提供了有力支撑。

## Abstract (en)

*N*-Glycosylation of proteins, an important post-translational modification in eukaryotic cells, plays an essential role in the regulation of cell adhesion, migration, signal transduction, and apoptosis. Abnormal changes in protein glycosylation are closely related to the occurrence of many critical diseases, including diabetes, tumors, and neurological, kidney, and inflammatory diseases. A non-invasive type of liquid biopsy, urine sampling has the advantage of reducing the complexity of proteomic analysis. This facilitates the design of large-scale and continuous or multi-time point sampling strategies. However, the dynamic range of urinary protein abundance is relatively large, owing to individual differences and physiological conditions. Currently, there is a lack of specialized research on individual differences, physiological fluctuations, and physiological abundance ranges of urinary *N*-glycoproteins in large healthy populations. Therefore, it is difficult to accurately distinguish individual differences and normal physiological fluctuations from changes caused by disease; this poses a great challenge in disease marker research. Liquid chromatography-mass spectrometry (LC-MS) is an analytical technique widely used for the large-scale profiling of proteomes in biological systems, and the enrichment of *N*-glycopeptides is a prerequisite for their detection by MS.

In this study, we established an approach based on hydrophilic interaction chromatography (HILIC) by optimizing the activation, cleaning, and elution processes of the enrichment method, for instance through the optimization of particle size and solvent composition, and investigated the identification number, selectivity, and stability of *N*-glycoprotein/*N*-glycopeptide enrichment under different experimental conditions. We found that *N*-glycoproteins and *N*-glycopeptides were highly enriched in a trifluoroacetic acid system with 5-μm filling particles in the HILIC column. On this basis, we analyzed the levels of *N*-glycoproteins/*N*-glycopeptides in urine samples. The consistency of *N*-glycoprotein/*N*-glycopeptide levels in urine samples taken from the same healthy person for five consecutive days was investigated by correlation analysis. This analysis revealed that the urinary *N*-glycoproteome of the same healthy person was relatively stable over a short period of time. Next, urinary samples from 20 healthy male volunteers and 20 healthy female volunteers were enriched for *N*-glycoproteins/*N*-glycopeptides, which were profiled by MS through qualitative and quantitative analyses. Screening and functional analysis of differential proteins were then carried out. A total of 1016 *N*-glycoproteins and 2192 *N*-glycopeptides were identified in the mid-morning urine samples of the 40 healthy volunteers. A label-free quantitation strategy was used to investigate the fluctuation range of the physiologically abundant urinary *N*-glycopeptides. The abundance of urinary *N*-glycopeptides spanned across approximately five orders of magnitude. Subsequently, gender differences in the *N*-glycosylation levels of urinary proteins were also explored in healthy people. Functional analysis of the *N*-glycoproteins that exhibited gender differences in abundance was performed. Based on multivariate statistical analysis, 206 differentially expressed proteins (*p*<0.05, fold change (FC)> 4) were identified. In females, we found 175 significantly down-regulated *N*-glycoproteins and 31 significantly up-regulated *N*-glycoproteins with respect to males. The expression levels of *N*-glycopeptides between the two groups suggested a clear gender difference. To investigate the biological processes and functions of these proteins, gene ontology (GO) analysis was performed on the *N*-glycoproteins/*N*-glycopeptides differentially expressed between males and females. Metabolic pathway analysis was also carried out based on the kyoto encyclopedia of genes and genomes (KEGG). Differentially expressed *N*-glycoproteins were mostly associated with platelet degranulation, extracellular region, and ossification. The top three relevant pathways were glycan biosynthesis and metabolism, metabolism of cofactors and vitamins, and lipid metabolism. Overall, sex may be an important factor for urinary *N*-glycoproteome differences among normal individuals and should be considered in clinical applications. This study provides relevant information regarding the function and mechanisms of the urinary glycoproteome and the screening of clinical biomarkers.

*N*-糖基化修饰是一种普遍的生物学过程,在蛋白质的折叠、运输中都承担着重要的作用^[1]^。许多细胞生理功能及生物学过程对糖基化高度敏感^[2,3]^,如细胞识别和信号转导^[4]^、细胞黏附^[5]^、免疫应答^[6]^及细胞凋亡等。当*N*-糖基化修饰进程表现异常,多种疾病,包括神经性疾病^[7]^、糖尿病^[8]^、肾病^[9]^、肿瘤^[10,11]^及炎症疾病^[12,13]^等通常会被引发。因此,深入考察*N*-糖蛋白/*N*-糖肽对疾病预防、诊断^[14,15]^、分期和疗效追踪评价有显著的临床参考意义。

尿液是发现疾病生物标志物、监测身体健康状态及临床诊断的常用生物样本。来源于肾小球滤过及泌尿系统分泌的尿液蛋白质组的变化可以反映人体的生理、病理状态^[16]^。因为血液受机体的稳态调节,在*N*-糖蛋白质组层面发生的早期细微变化一般只能短暂存在即可能被清除,然而尿液作为人体废弃或有害物质的集中储存场所,则刚好可以接收、储存并累积机体中的生理及病理因素,并不受机体稳态调节影响,因此尿液在*N*-糖蛋白质组上的研究意义和价值无法被忽略。除泌尿系统外,尿液蛋白质组研究对消化系统、心血管和内分泌系统等的快速诊断、疗效观察、预后评估以及人群健康保健都有重要价值。同时尿液取样方式完全无侵入性,可重复多次取样,且由于尿液具有更低的生物复杂性,易于分析而被广泛研究^[17]^。但尿液中糖蛋白质组的生理丰度会因个体间差异和生理条件的变化而波动^[18]^,目前尚无针对健康人群尿液中*N*-糖蛋白生理丰度范围的研究。因此,难以判断临床疾病生物标志物研究中所发现的糖蛋白差异究竟是来自于正常生理波动、个体间差异还是疾病导致的变化,对后期大规模样本验证提出极大挑战^[18]^。

在复杂生物样本中,糖基化肽段丰度较低^[19,20,21]^(在全部蛋白质酶解肽段的占比不高于5%),同时其具有高异质性^[8,22]^及较宽的动态范围,且糖肽离子化效率较低,因此进行*N*-糖蛋白/*N*-糖肽的高效分离富集是实现*N*-糖蛋白质组深度覆盖的重要前提^[23]^。目前*N*-糖蛋白/*N*-糖肽的富集方法主要包括凝集素纯化法、酰肼化学法、硼酸法和亲水相互作用色谱法等^[21,24]^。凝集素纯化法主要用于分析具有特定类型聚糖结构的糖蛋白/糖肽,聚糖结构覆盖率低^[25]^。酰肼化学法^[26]^反应时间过长,糖链结构易被破坏。硼酸法^[9,27]^因糖型不同会产生较大富集差别。亲水相互作用色谱法(hydrophilic interaction chromatography, HILIC)^[28,29,30]^对聚糖的俘获呈现广谱性、强保留性以及较高稳定性,同时HILIC高效快捷^[21]^,具有不易破坏糖链、溶剂温和以及易兼容质谱等优点,已经被广泛应用于生物样本中*N*-糖肽的富集^[11,31,32]^。

本研究从HILIC填料粒径和缓冲溶液两方面优化了HILIC富集条件,并考察评价了*N*-糖肽富集的选择性与稳定性。之后,我们选取了20例健康男性志愿者和20例健康女性志愿者的中段晨尿对*N*-糖蛋白/*N*-糖肽进行了定性、定量及功能分析,并对健康人群男性与女性尿蛋白*N*-糖基化水平的性别差异探索研究。在此基础上采用非标定量策略对同一个体多时间点及不同个体的尿液*N*-糖肽的生理丰度波动进行了考察。本工作为基于尿液糖蛋白质组学的功能与机制研究和临床生物标志物筛选奠定了基础^[8]^。

## 1 实验部分

### 1.1 仪器和试剂

高速离心机、NanoDrop 2000 C超微量分光光度计与EASY-nLC 1000 LC-MS/MS纳升级液相色谱-串联Q Exactive质谱仪购自Thermo Scientific(美国); Sartorius CPA225D分析天平购自赛多利斯公司(德国);碳酸氢铵(ABC)、丙酮购自国药集团化学试剂有限公司;尿素购自VWR公司(美国);二硫苏糖醇(DTT)、碘乙酰胺(IAA)、甲酸(FA)和三氟乙酸(TFA)购自Sigma-Aldrich公司(美国); C_8_滤膜购自3M公司(美国);肽*N*-糖酰胺酶F (PNGase F, 500 U/μL)购自New England Biolabs(英国);胰蛋白酶(trypsin)购自Promoga公司(美国); $H_{2}^{18}$ O(纯度≥98%)购自上海化工研究院;HPLC级乙腈(ACN)购自Merck公司(德国),化学试剂纯度为分析级或HPLC级。过滤器辅助的样品制备(filter-aided sample preparation, FASP) 离心管购自Millipore(美国);去离子水由Millipore纯水仪制备;健康人中段泌尿道晨尿由青年志愿者提供。

### 1.2 实验方法

1.2.1 丙酮提取尿蛋白

取20 mL尿液在4 ℃条件下以3000 g离心30 min,随后以12000 g离心30 min,提取上清液。将上清液均分至2个高速离心管中,再加入其3倍体积的预冷丙酮(-20 ℃),混合均匀后放入-20 ℃冰箱中沉淀4 h。取出以后将样本在4 ℃以12000 g离心30 min,取管中的沉淀加入400 μL 8 mol/L尿素溶液溶解尿蛋白,200 W超声后离心弃去沉淀,取上清液备用。

1.2.2 FASP酶解

将所提取的尿蛋白转移至30 kD超滤管中,以14000 g离心10 min。在尿蛋白样本中加入DTT保持终浓度为10 mmol/L,于37 ℃变性反应4 h。再向样本中加入200 μL 50 mmol/L的IAA,于室温避光处孵育40 min,随后加入200 μL 50 mmol/L的ABC溶液(pH=8.4)清洗置换溶液体系。在尿蛋白中加入0.5 mg/mL胰蛋白酶(胰蛋白酶与蛋白质的质量比为1:100)于37 ℃酶切,共加入两次,第一次酶切时间为12 h,第二次为4 h。酶切后14000 g离心10 min收集肽段,并测定浓度,然后移取80 μg肽段,在45 ℃下离心浓缩至3~5 μL,剩余肽段置于-80 ℃冰箱保存备用。

1.2.3 HILIC富集*N*-糖肽

每80 μg肽段对应称取5 mg粒径为5 μm的HILIC填料,首先加入100 μL 0.5% (v/v) TFA活化10 min后,再用0.5% (v/v) FA活化30 min,每次100 μL,清洗3次。然后用TFA体系清洗液TFA-H_2_O-ACN (1:19:80, v/v/v)平衡30 min,向肽段中加入HILIC填料混合液后,30 ℃振荡孵化2 h,样品全部转移至装填2层C_8_膜的tip头内,加入80 μL清洗液清洗3次后更换离心管洗脱*N*-糖肽。洗脱液TFA-H_2_O-ACN (1:79:20, v/v/v)洗脱3次,每次80 μL。45 ℃下离心浓缩后用20 μL 25 mmol/L ABC/ $H_{2}^{18}$ O复溶肽段,测定pH在7.5左右,加入100 U PNGase F, 37 ℃水浴酶切16 h。然后分别用80 μL的100%ACN、50% (v/v) ACN和0.1% (v/v) TFA活化3层C_18_膜,取3.5 μL酶切物混合于80 μL 0.1% (v/v) FA中脱盐,其余保留至-80 ℃冰箱贮存。样品反复3次移取至tip头中,注射器按压,80 μL 0.1% (v/v) FA清洗3次,50% ACN/0.1% FA (v/v)清洗2次。45 ℃下离心浓缩,进行LC-MS/MS鉴定。

### 1.3 LC-MS/MS分析方法

采用C_18_反相毛细管柱(120 mm×150 μm, 1.9 μm),流动相A和B分别为含0.1% (v/v) FA的水溶液和含0.1% (v/v) FA的乙腈溶液,以0.6 μL/min流速洗脱样本90 min。洗脱梯度为8~60 min, 10%B~30%B; 60~79 min, 30%B~42%B; 79~80 min, 42%B~95%B; 80~85 min, 95%B。在正离子模式下采集谱图,一级质谱扫描质荷比范围是300~1400,分辨率为70000。二级质谱分辨率为17500,隔离窗口(*m/z*)为3,动态排除时间是15 s。

### 1.4 数据分析

质谱数据在Maxquant 1.5.2.8软件中以Uniprot_human(2015.7, 20207条肽段)为蛋白数据库进行检索。检索参数设置如下:蛋白水解酶为胰蛋白酶,糖蛋白鉴定最大漏切数目为两个,每个肽段最大修饰数设为5个,最大电荷数设为7,可变修饰包括蛋白质N末端乙酰化、甲硫氨酸氧化修饰和脱酰胺^18^O位点修饰,固定修饰包括半胱氨酸烷基化修饰。母离子的质量容差最大设置为0.00045% (w/w),二级碎片离子的质量容差最大为0.002% (w/w),蛋白质及肽谱匹配(peptide-spectrum match, PSM)的假阳性率(false discovery rate, FDR)设为1%。

为了筛选尿液中与性别相关的*N*-糖蛋白,对*N*-糖肽进行统计学分析。先对每个*N*-糖肽的丰度值进行转换后进行统计学检验,再以*p*<0.05和倍数变化(fold change, FC)>4的标准筛选出存在性别特异性的差异蛋白,随后对筛选出的*N*-糖蛋白进行聚类分析,定量分析不同性别组蛋白质表达水平的差异。通过Uniprot及DAVID对具有显著表达差异的蛋白(前景蛋白)进行细胞定位、生物学过程及功能的基因本体(gene ontology, GO)注释和富集分析,同时通过京都基因与基因组百科全书(kyoto encyclopedia of genes and genomes, KEGG)数据库进行代谢通路分析。

## 2 结果与讨论

### 2.1 方法优化及评价

在亲水相互作用色谱法的基础上,优化条件使HILIC富集具有更高选择性和稳定性对于糖蛋白质组研究至关重要。当前*N*-糖肽提取富集的过程主要包括提取、酶切和富集3个步骤。我们对富集过程中HILIC填料的活化、孵育、清洗及特异性洗脱步骤进行了优化,主要涉及对填料粒径与缓冲溶液等条件的深入考察。

2.1.1 HILIC填料粒径筛选

评估了1.5 μm、3 μm和5 μm 3种粒径的填料对*N*-糖肽富集的效果。在FA富集体系下,清洗液为FA-H_2_O-ACN (5:15:80, v/v/v),洗脱过程包括80 μL 0.5% (v/v) FA洗脱3次,80 μL FA-H_2_O-ACN (0.5:94.5:5, v/v/v)洗脱1次。结果如下:使用1.5 μm粒径进行HILIC富集,*N*-糖蛋白及*N*-糖肽平均鉴定量为512和865,选择性为64.08%;使用3 μm粒径填料,*N*-糖蛋白及*N*-糖肽平均鉴定量分别为549和942,选择性为73.92%;使用5 μm粒径填料,*N*-糖蛋白及*N*-糖肽平均鉴定量分别为575和1008,选择性为78.27%。HILIC富集法主要基于*N*-糖肽在流动相与HILIC固定相之间“富水层”分配系数不同而实现分离^[33]^。如图1所示,采用5 μm粒径的HILIC填料进行富集显示出更高的*N*-糖蛋白及*N*-糖肽鉴定量,且5 μm粒径下的富集选择性(*N*-糖肽鉴定量/肽段鉴定量)及稳定性也最高,因此选择5 μm粒径并进行下一步条件优化。

2.1.2 缓冲体系优化

为了评价不同缓冲体系的富集效果,我们主要采用TFA与FA两种缓冲体系进行对比考察,涉及ACN浓度、酸浓度和洗脱次数等参数的评估,分别对两种缓冲体系下的清洗液和洗脱液及洗脱步骤进行了优化,并采用*N*-糖蛋白、*N*-糖肽、肽段鉴定量及选择性作为评价指标。在TFA体系下对一名健康志愿者的6例晨尿进行质谱分析,平均鉴定到1142条*N*-糖肽,621个*N*-糖蛋白,选择性为78.80%。在FA体系下从相同样本中鉴定到812条*N*-糖肽,478个*N*-糖蛋白,平均选择性为58.18%。如图2所示,TFA体系下的*N*-糖蛋白和*N*-糖肽的平均鉴定量均高于FA体系,且TFA体系下数据的平均相对标准偏差(RSD)值远低于FA体系。同时TFA体系下具有更高的选择性,说明TFA体系下*N*-糖蛋白/*N*-糖肽鉴定覆盖率更高,方法更稳定。研究显示,相比于非离子对试剂,TFA作为离子对试剂通常对*N*-糖肽具有更高的特异选择性,该实验结果与文献^[31,34]^相符。

**图 2:**
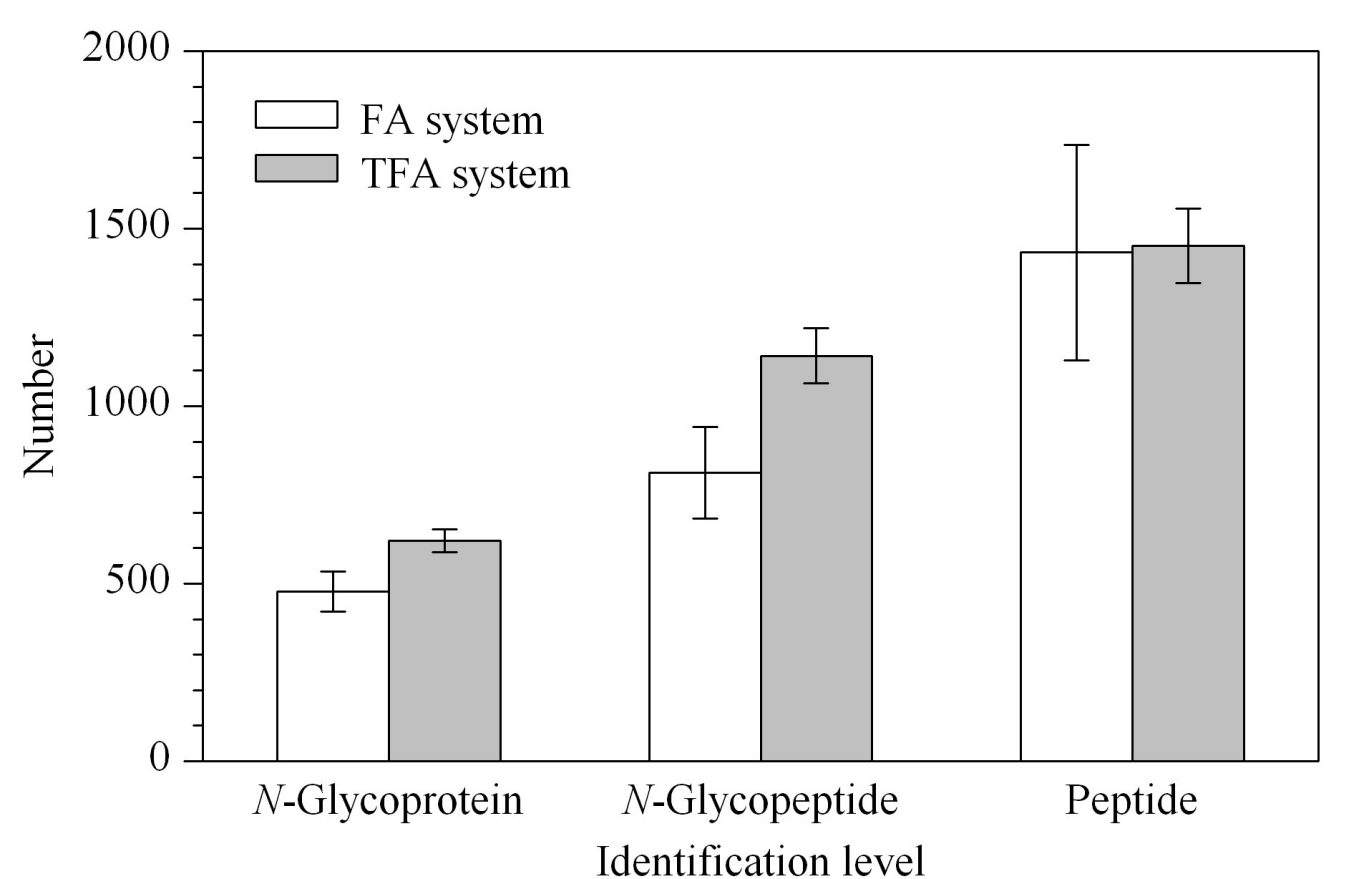
同一健康志愿者样本在2种富集体系下的质谱鉴定水平(*n*=6)

### 2.2 同一健康人连续时间点样本*N*-糖蛋白质组分析

为了观察健康人随时间推移的*N*-糖蛋白质组表达水平变化情况,本研究采集了同一健康青年志愿者连续5天晨尿的*N*-糖蛋白质组信息。质谱分析共鉴定出665个*N*-糖蛋白,1238条*N*-糖肽,可定位到1239个*N*-糖基化位点。根据样本两两比较的相关性分析结果,如图3显示,样本间*N*-糖蛋白质组Spearman平均相关性系数为0.901,波动范围在0.854~0.942,具有较高相关性,说明同一健康人短时间内尿液*N*-糖蛋白质组的生理波动比较稳定。

**图 3:**
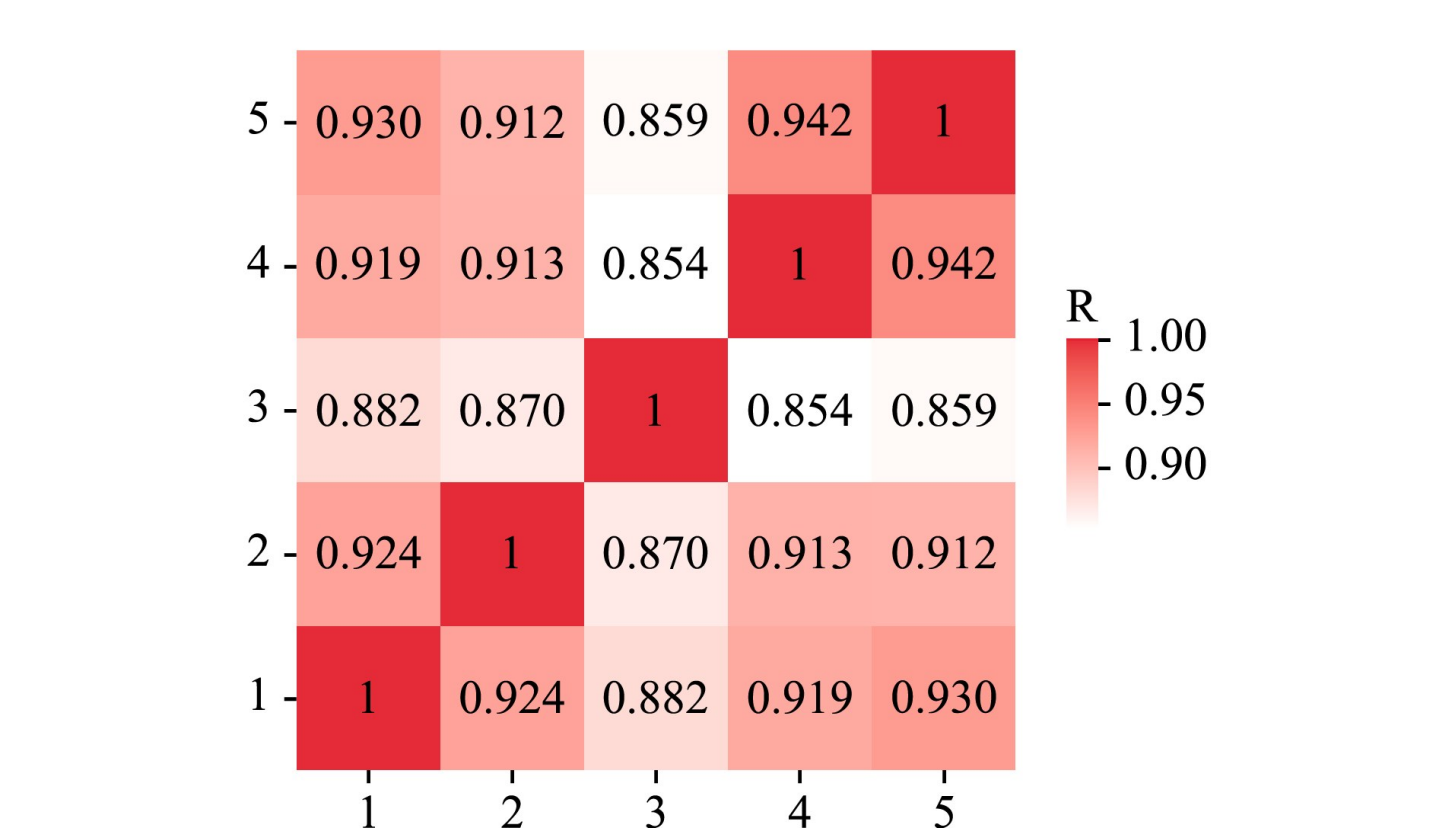
同一健康志愿者连续5天尿样相关性分析

### 2.3 健康人样本糖蛋白质组学分析

本实验在经过伦理委员会审查与批准后进行志愿者招募,参与的受试志愿者均已知悉实验目的且签署知情同意书。本研究采用LC-MS/MS对40例健康志愿者(男性20例,女性20例)尿样进行个体化*N*-糖蛋白质组鉴定,采用非标定量法对每例尿样中的*N*-糖肽丰度进行定量,并进行后续差异蛋白筛选及功能分析。

2.3.1 尿液*N*-糖蛋白质组分析

实验从40例尿样中共鉴定到1016个*N*-糖蛋白、2192条*N*-糖肽和2194个*N*-糖基化位点。根据40例尿样的Spearman相关性分析结果(见图4a),40例健康人尿样平均相关性系数为0.475,其中20例男性和20例女性尿样平均相关性系数分别为0.544和0.460(见图4b和4c),男性样本相关性高于女性,也说明尿液中*N*-糖蛋白质组的生理丰度可能会因存在生理方面差异、个体间差异(如性别差异)而产生波动。*N*-糖蛋白和*N*-糖基化位点鉴定量累积曲线如图5所示,其累积鉴定量分别在样本数量达到14例与20例之后趋于饱和,没有继续显著上涨趋势。这为40例样本的*N*-糖蛋白鉴定及后续定量分析奠定基础。

**图 4:**
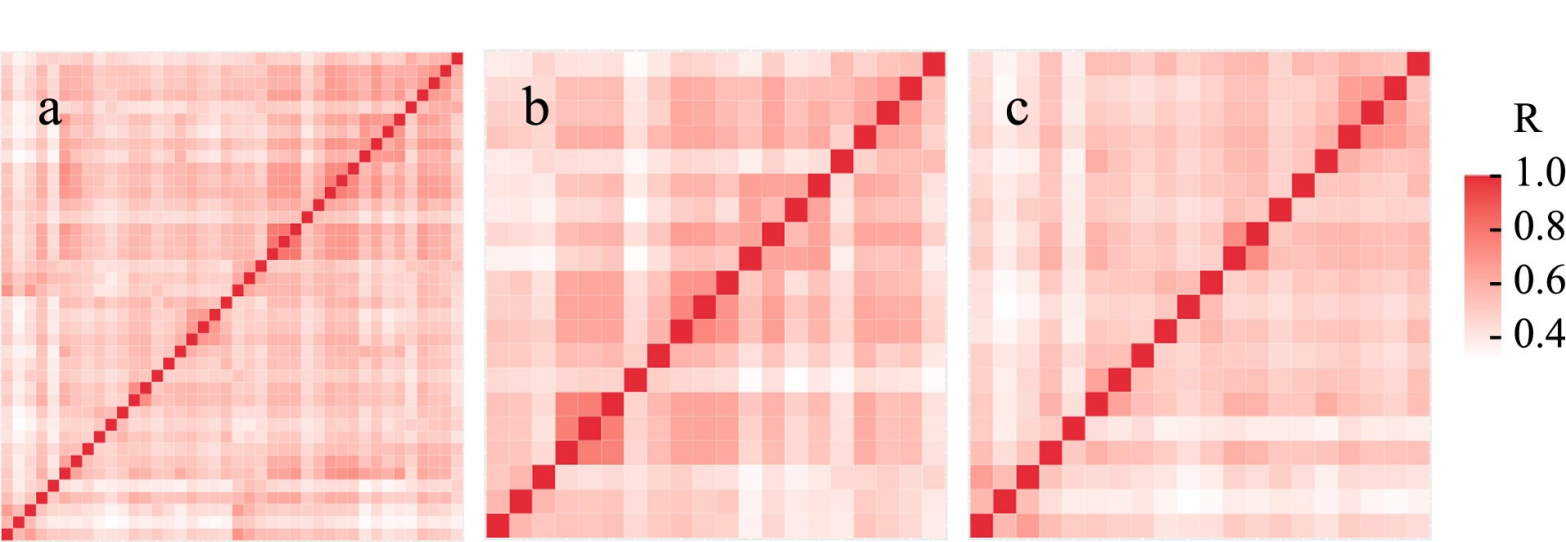
不同健康志愿者尿样的相关性分析

**图 5:**
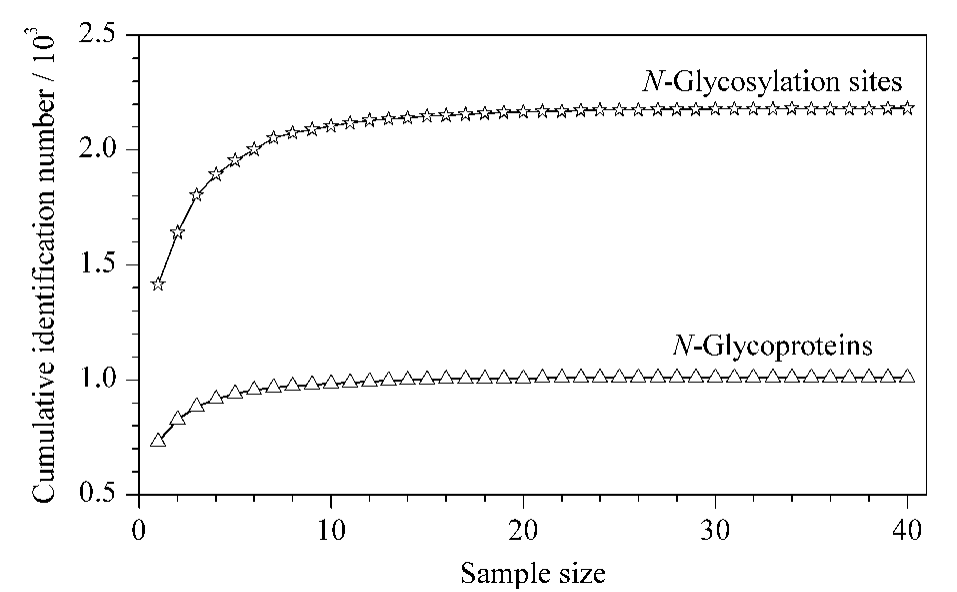
不同样本量下*N*-糖蛋白及*N*-糖基化位点累积鉴定量曲线图

2.3.2 尿液*N*-糖蛋白定量解析

为满足大规模人群尿液*N*-糖蛋白分析的需求,本研究采用了非标定量法。为了更直观地获取健康人尿液*N*-糖蛋白质组动态变化范围,我们对40例健康个体的*N*-糖肽丰度分布情况进行分析,如图6所示,横坐标代表非标定量所得*N*-糖肽丰度的对数值,纵坐标代表不同丰度区间内*N*-糖肽的比例分布情况。*N*-糖肽的丰度动态范围跨越了5个数量级,并且右半部分曲线比左侧走势更加平缓,*N*-糖肽丰度为正偏态分布。这说明尿液*N*-糖肽的丰度跨度范围较大,并且该富集鉴定方法可有效覆盖较多低丰度的*N*-糖肽,对于生物标志物研究至关重要。*N*-糖肽在低-中-高的丰度范围内所占比例展现为先增加后减少的变化趋势,反映了健康人尿液中*N*-糖肽丰度的总体分布规律。

**图 6:**
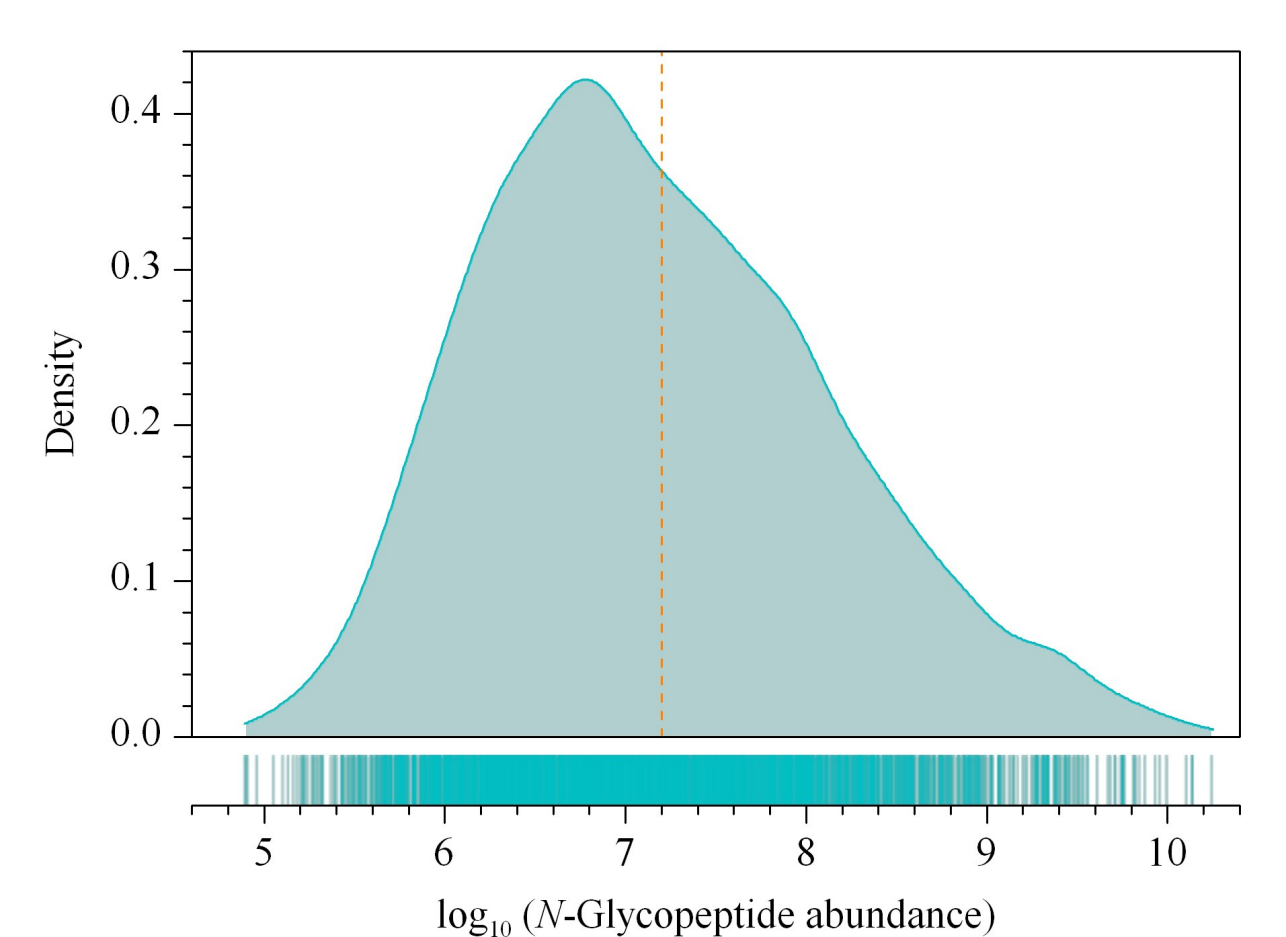
40例健康志愿者尿样*N*-糖肽丰度的总体分布规律图

2.3.3 *N*-糖蛋白功能注释与通路分析

为了初步了解富集到的*N*-糖蛋白的功能与相关通路,我们对全部*N*-糖蛋白进行了GO分析和KEGG通路富集分析。GO分析主要包括生物学过程、分子功能及亚细胞定位3个方面。生物学过程中,*N*-糖蛋白中占比较高的为血管生成、丝裂原活化蛋白激酶(mitogen-activated protein kinase, MAPK)级联、血小板脱粒、细胞形态发生及RNA聚合酶II对转录的负调控等。细胞亚细胞定位为高尔基体膜、细胞外区域、质膜及胞外空间等。主要涉及的细胞功能有钙离子结合、丝氨酸型内肽酶活性、病毒受体活性、跨膜信号受体活性及细胞外基质结构组成等(见附图1a,http://www.chrom-China.com)。其中显著富集的前5个GO条目是血管生成、细胞外区域、病毒受体活性、高尔基体及丝氨酸型内肽酶活性(见附图1b,http://www.chrom-China.com)。KEGG富集分析中,与*N*-糖蛋白相关的疾病主要有心血管疾病和免疫疾病等,涉及免疫系统、发育与再生等生物系统等(见附图2a, http://www.chrom-China.com)。其中显著富集到的5个通路为细胞黏附分子、补体和凝血级联、溶酶体、轴突导向及细胞因子-细胞因子受体相互作用(见附图2b, http://www.chrom-China.com)。

**图 1:**
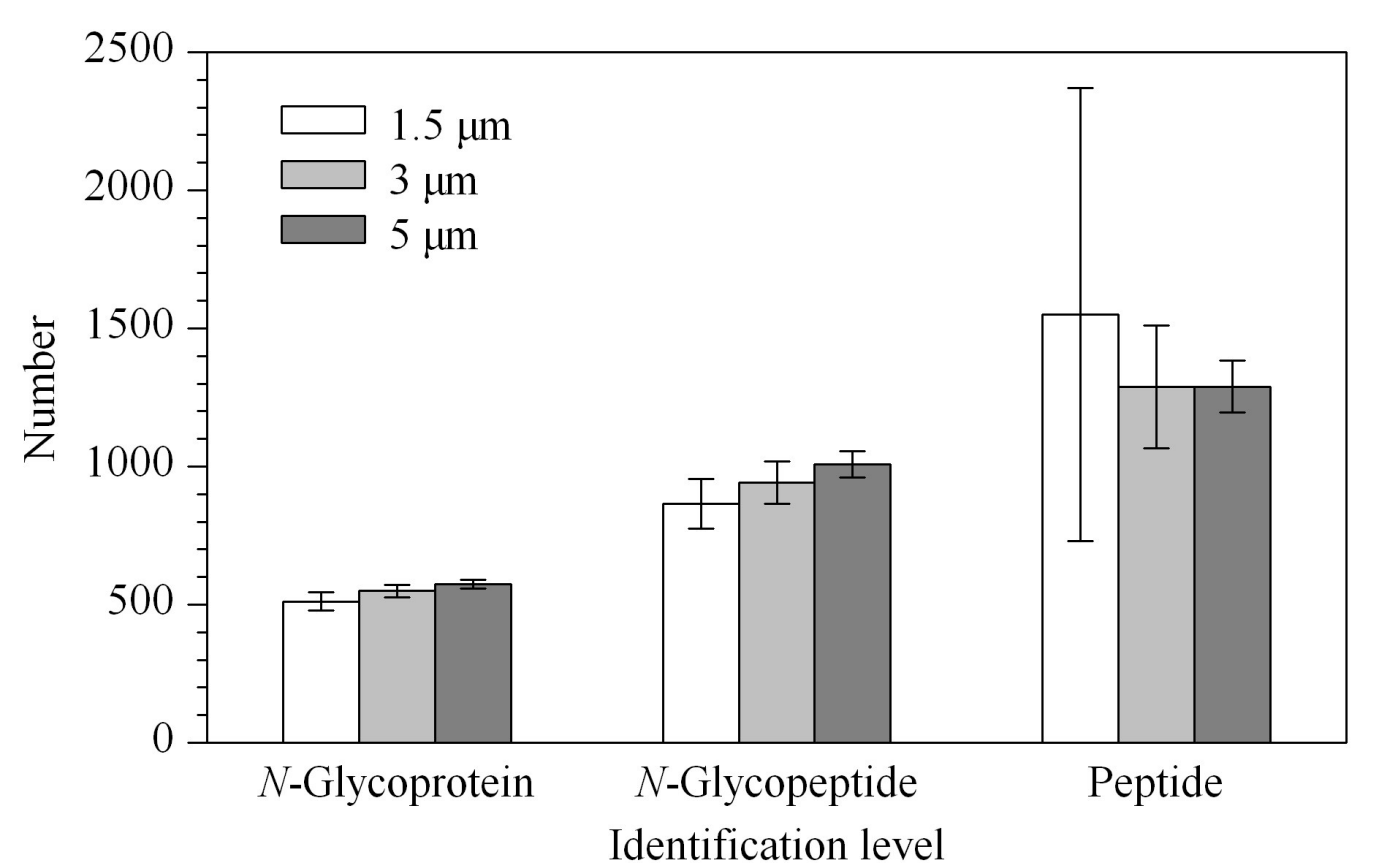
同一健康志愿者样本使用3种不同粒径HILIC 填料富集后的质谱鉴定规模(*n*=3)

2.3.4 男女人群差异蛋白分析

通过分析40例健康志愿者尿液*N*-糖蛋白质的表达情况,我们共鉴定到206个差异表达蛋白。如图7a所示,横坐标为女性与男性*N*-糖肽定量值倍数比的对数值,纵坐标表示*p*值的负对数。其中蓝色代表相比于男性,女性显示下调的*N*-糖肽,红色代表相比于男性,女性显示上调的*N*-糖肽。女性人群有175个*N*-糖蛋白相比于男性明显下调,有31个*N*-糖蛋白比男性显著上调。接下来对筛选到的差异*N*-糖蛋白的表达水平进行聚类分析。如热图7b显示,两者*N*-糖肽的表达量显示出明显的性别差异,说明性别可能是正常个体尿液中*N*-糖蛋白质组存在差异的一项重要因素,在临床诊断及生物标志物的筛选中应予以考虑。

**图 7:**
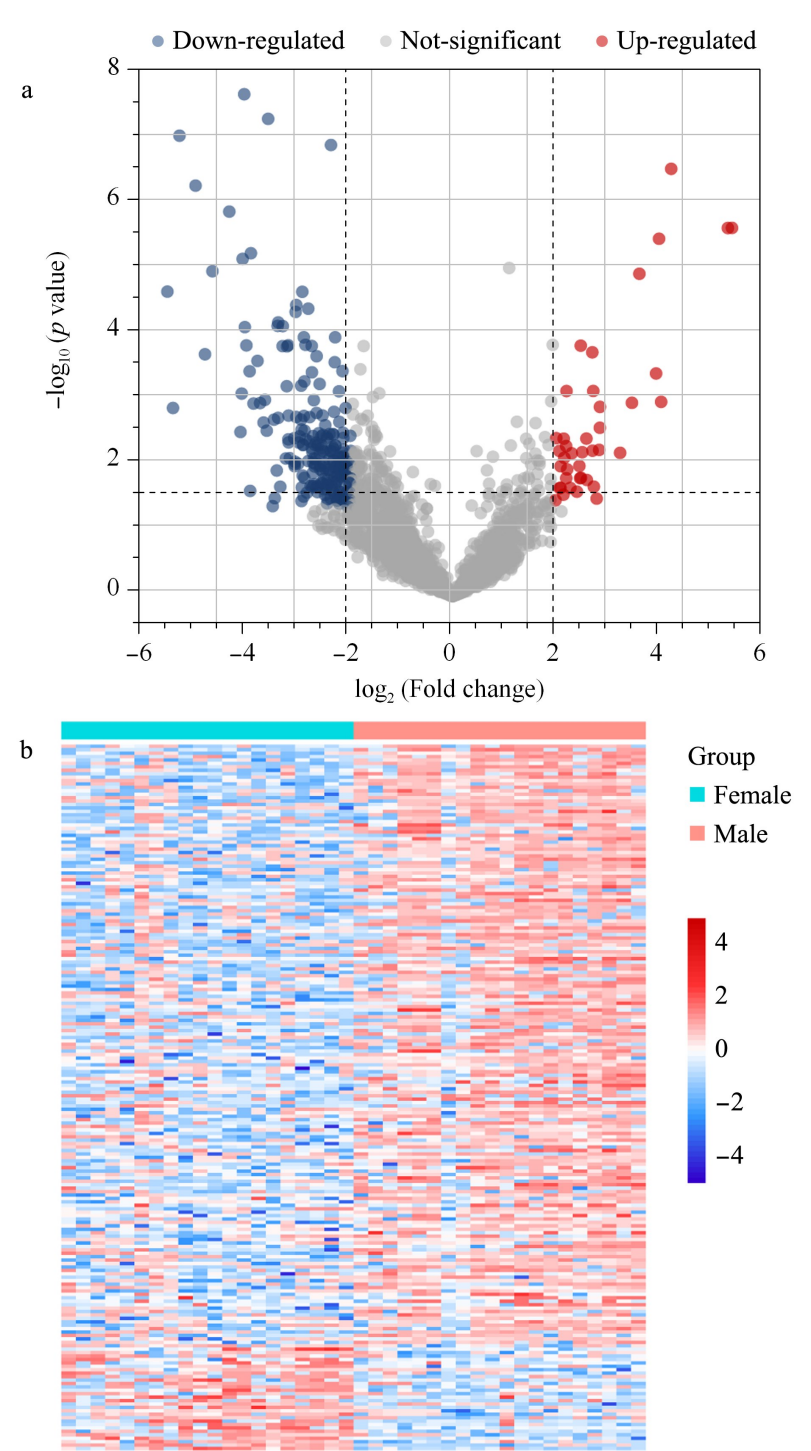
(a)依赖于性别差异表达的*N*-糖蛋白的火山图 与(b)差异表达水平热图

2.3.5 *N*-糖蛋白功能注释与通路分析

为研究男女人群中存在表达差异的*N*-糖蛋白在生物学过程、分子功能及亚细胞定位方面的差异,我们对其进行了GO分析。如图8a所示,差异蛋白中占比高的生物学过程包括血小板脱粒、血管生成、骨化、成骨细胞分化与RNA聚合酶II对转录的负调控等。差异蛋白定位的主要亚细胞区域包括细胞外区域、高尔基体膜、细胞质膜与细胞质等。分子功能分析发现钙离子结合、丝氨酸型内肽酶活性、病毒受体活性、细胞外基质结构构成与蛋白酶结合等与差异蛋白密切相关。其中显著富集到的前5个GO条目为血小板脱粒、细胞外区域、骨化、急性炎症反应的正调控及细胞外基质结构组成等(见图8b)。

**图 8:**
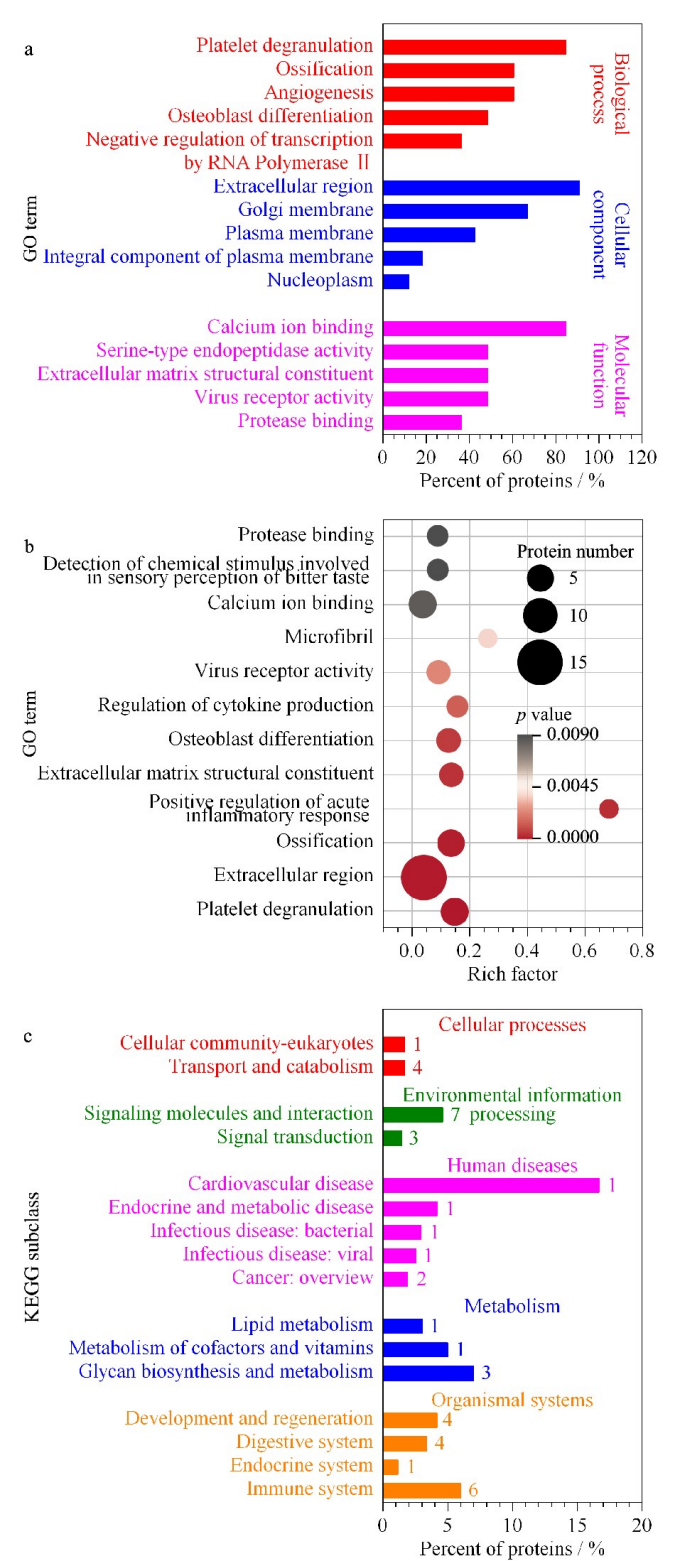
基于差异表达的*N*-糖蛋白的功能注释与通路分析

此外根据KEGG数据库分析,显著富集到的代谢通路涉及130个*N*-糖蛋白。在差异蛋白中占比较高的前3个通路为聚糖的生物合成与代谢、辅助因子和维生素代谢及脂质代谢。疾病方面,差异蛋白在心血管疾病、内分泌和代谢疾病、细菌或病毒性传染病及癌症疾病中具有较高比例。生物系统里,在免疫系统、发育与再生与消化系统等占比更丰富(见图8c)。其中显著富集到的前3个代谢通路为细胞黏附分子、补体和凝血级联反应及其他糖降解。

## 3 结论

综上所述,本研究在优化HILIC富集条件的基础上,通过5例同一健康人的多时间点尿液样本考察了短时间内尿液*N*-糖蛋白质组的生理性波动情况。并通过对20例健康男性和20例健康女性尿液样本的*N*-糖蛋白/*N*-糖肽的定性、定量分析,考察了健康人群尿液*N*-糖蛋白/*N*-糖肽的定量范围。进而对健康人群尿蛋白*N*-糖基化水平进行了性别差异研究,筛选到206个男性和女性的差异*N*-糖蛋白。通过对差异*N*-糖蛋白的功能注释及通路解析,挖掘了男女健康人群尿蛋白*N*-糖基化水平呈现显著变化的分子信息,提示性别差异作为一个影响因素在基于尿液糖蛋白质组的疾病标志物研究中需给以重视。
